# IMG/M-HMP: A Metagenome Comparative Analysis System for the Human Microbiome Project

**DOI:** 10.1371/journal.pone.0040151

**Published:** 2012-07-05

**Authors:** Victor M. Markowitz, I-Min A. Chen, Ken Chu, Ernest Szeto, Krishna Palaniappan, Biju Jacob, Anna Ratner, Konstantinos Liolios, Ioanna Pagani, Marcel Huntemann, Konstantinos Mavromatis, Natalia N. Ivanova, Nikos C. Kyrpides

**Affiliations:** 1 Biological Data Management and Technology Center, Lawrence Berkeley National Laboratory, Berkeley, California, United States of America; 2 Microbial Genomics and Metagenomics Program, Department of Energy Joint Genome Institute, Walnut Creek, California, United States of America; Miami University, United States of America

## Abstract

The Integrated Microbial Genomes and Metagenomes (IMG/M) resource is a data management system that supports the analysis of sequence data from microbial communities in the integrated context of all publicly available draft and complete genomes from the three domains of life as well as a large number of plasmids and viruses. IMG/M currently contains thousands of genomes and metagenome samples with billions of genes. IMG/M-HMP is an IMG/M data mart serving the US National Institutes of Health (NIH) Human Microbiome Project (HMP), focussed on HMP generated metagenome datasets, and is one of the central resources provided from the HMP Data Analysis and Coordination Center (DACC). IMG/M-HMP is available at http://www.hmpdacc-resources.org/imgm_hmp/.

## Introduction

The Integrated Microbial Genomes and Metagenomes (**IMG/M**) system provides support for comparative analysis of metagenome sequence data generated by sequencing microbial communities (microbiomes), in the integrated context of a continuously expanding universe of genome and metagenome datasets generated worldwide. Assembled or unassembled metagenome datasets generated using Illumina sequencing platform are processed by JGI’s metagenome annotation pipeline [1] before inclusion into IMG/M [2]. Unassembled reads undergo an additional quality control step which includes quality trimming, low complexity region detection and masking, as well as removal of technical replicates. Subsequently, both assembled and unassembled sequences are annotated by the same pipeline, which detects CRISPR repeats [3], non-coding RNAs, and protein-coding genes (CDSs). RNAs are predicted using tRNA-Scan-SE-1.23 [4] for tRNAs, and in-house developed HMM models for rRNAs, while the CDSs are identified using a combination of *ab initio* gene prediction tools, Prodigal [5], Metagene [6], MetaGenemark [7], and FragGeneScan [8]. Conflicting gene predictions are consolidated using a weighted schema based on the performance of each method on simulated datasets, with one final gene model generated for each region.

Analysis of metagenome data includes determining the phylogenetic composition and functional or metabolic potential within individual microbiomes, as well as comparisons across microbiomes. IMG/M provides support for such analysis by integrating metagenome datasets with isolate microbial genomes from the Integrated Microbial Genome (IMG) system [9]. IMG integrates draft and complete microbial genomes from all three domains of life with a large number of plasmids and viruses. Similar to IMG, IMG/M records the primary sequence information for isolate genomes and metagenomes, their organization in scaffolds and/or contigs, as well as computationally predicted protein-coding sequences and RNA-coding genes. Protein-coding genes are characterized in terms of additional annotations, such as conserved motifs and domains, signal peptides, transmembrane helices, pathways and orthology relationships, which may serve as an indication of their functions. These annotations are based on diverse data sources, such as COG clusters and functional categories [10], Pfam [11], TIGRfam and TIGR role categories [12], InterPro domains [13], and KEGG Orthology (KO) terms and pathways [14].

Metagenome datasets are first included into IMG/M’s “Expert Review” version**,** IMG/M ER, which allows scientists to employ IMG/M’s annotation pipeline as well as review and curate the functional annotation of metagenomes in the context of IMG/M’s reference genomes and public metagenomes prior to public release of their datasets. **IMG/M-HMP** is an IMG/M ER **data mart** focussed on metagenome datasets produced by the US National Institute of Health (NIH) Human Microbiome Project (HMP) and is part of the HMP Data Analysis and Coordination Center (DACC). HMP aims to study the role of microbial communities associated with human body sites, such as nasal passages, oral cavities, skin, gastrointestinal and urogenital tracts, in human health [15]. In order to achieve this goal, HMP has embarked on sequencing a large number of reference genomes associated with human hosts [16] and metagenome samples collected from carefully screened and phenotyped human subjects [17]. HMP’s DACC hosts datasets generated by HMP and various computational tools and resources, such as the HMP reference strain catalog (http://www.hmpdacc.org/).

## Results and Discussion

### HMP Data and Organization

IMG/M-HMP contains **748 metagenome datasets** generated as part of the HMP initiative by sequencing samples collected from various body sites (airways, gastrointestinal, oral, skin, urogenital). This first release of IMG/M-HMP includes only the subset of assembled metagenome sequences on which a total of **80 million** protein coding genes have been predicted [17]. Metagenome datasets are integrated with publicly available bacterial, archaeal, eukaryotic, and viral genomes, including reference genomes sequenced as part of the HMP initiative.

**Figure 1:**
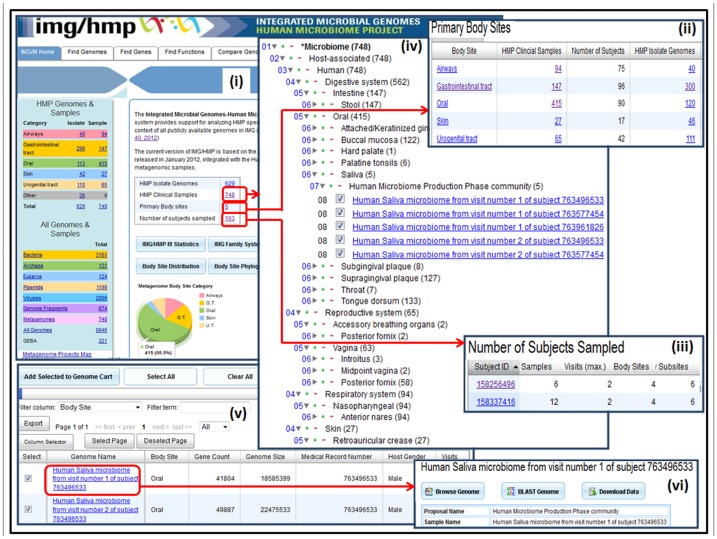
Grouping and Classification of metagenome datasets in IMG/M HMP.

HMP genomes and metagenomes in IMG/M-HMP are grouped both by body site category and by taxonomy, as shown in the left upper and lower panes of Figure 1(i). Metagenome datasets are also grouped according to the primary body site and human subjects sampled, as shown in Figures 1(ii) and 1(iii), respectively. The names and classification of metagenome datasets in IMG/M-HMP are curated in GOLD [18] following a five-tiered classification system [19]. This classification scheme underlies the organization of metagenome datasets in IMG/M in general and IMG/M-HMP in particular, as illustrated in Figure 1(iv). Similar to the phylogenetic classification of isolate genomes, the classification of metagenomes is a critical element for conducting metagenome comparative analysis in a rapidly growing universe of metagenome datasets. Thus, metagenome datasets are organized in three main *ecosystem classes*, *environmental*, *host associated*, and *engineered* classes, then further divided in subclasses characterized by *ecosystem categories* (e.g., arthropoda, human, mammals, plants for host associated metagenomes), *ecosystem type* (e.g. digestive system, reproductive system, respiratory system, skin), *ecosystem subtype* (e.g., oral, intestine), and *specific ecosystem* (e.g., hard palate, palatine tonsils, saliva).

Metagenome datasets in IMG/M HMP can be selected using several browse and search tools [2], as well as using predefined groupings and classification discussed above and illustrated in Figure 1. Selected metagenome datasets are displayed as lists with each dataset associated with *medical record number*, *host gender* and *visits* metadata, as illustrated in Figure 1(v). Datasets of interest from the lists can be included into a “**Genome Cart**” for further analysis.

Individual metagenomes can be explored using the “**Metagenome Details**” page which provides a variety of tools for browsing, searching genes, or downloading metagenome datasets (Figure 1(vi)). This page also provides information (metadata) on the metagenome together with various statistics of interest, such as the number of genes that are associated with KEGG, COG, Pfam, InterPro or enzyme information.

### Comparative Analysis Tools

IMG/M-HMP’s front page provides three comparative analysis “workflows” of HMP metagenome datasets for estimating taxonomic composition of individual samples as well as predefined sample aggregates grouped by sampled body sites, and analysing them in the context of reference genomes grouped according to their taxonomy and isolation source (body site category).

**Figure 2:**
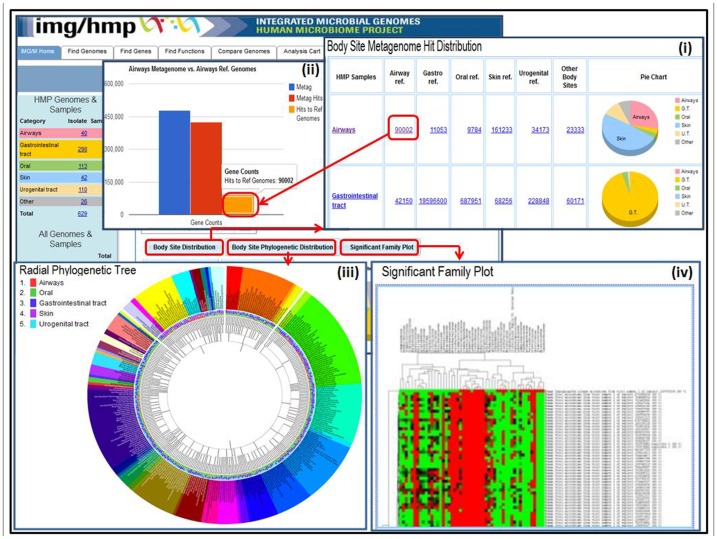
Distribution of genes of microbiomes grouped taxonomically and by habitat according to their best BLASTp hit to reference genomes.

The “**Body Sites Distribution**” (Figure 2(i)) shows the distribution of best BLASTp hits of the genes in the aggregate metagenome samples (grouped by body site) against the genes of the reference genomes grouped according to the body site category from which they were isolated. For example, there are 90,002 genes across all airways samples (from a total of 476,963 genes) that have their best BLASTp hits to some of the 286,127 genes of reference genomes isolated from human airways, as illustrated in Figure 2(ii).

As expected, in most cases the aggregate genes of the samples grouped by each of the primary body sites, had the highest percentage of their best BLAST hits against the genes of reference genomes isolated from the same body site (Table 1). For example 94.7% of the genes from the gastro-intestinal (GI) tract samples, have their best BLAST hits to genes of reference genomes isolated from GI tract. In a similar manner 73% of the genes from the urinary tract (UT) samples, have their best BLAST hits to genes of reference genomes isolated from UT. The only exception to this observation are the genes from the airway samples, most of which (48.9%) have their best BLAST hits to genes from reference genomes isolated from skin, and only 27% to genes from reference genomes isolated from airways. This may be explained by the fact that the majority of the airway samples have been collected from the anterior nares, whereas the majority of reference genomes classified into “Airways” category have been isolated from lower airways. Anterior nares are characterized by the presence of squamous epithelium which is an environment more similar to the skin than to lower airways covered with ciliated mucosa. Overall, the results of this type of data comparisons are heavily dependent on the quality of the metadata available for reference genomes: if the isolation site of the latter has not been properly documented in the original publication or accurately recorded in the database, then the observed results may be largely inaccurate and/or misleading.

**Table 1:** The percentage distribution of best blast hits of aggregate samples from each major body site run against reference isolate genomes grouped by each major body site.

	Body site of Reference Genomes					
Body Site of Sample	Airways	GI tract	Oral	Skin	UT tract	Other
Airways	27.3	3.4	3.0	48.9	10.4	7.1
GI tract	0.2	94.7	3.3	0.3	1.1	0.3
Oral	11.8	17.1	56.9	3.5	7.7	3
Skin	7.8	6.3	5.6	59.4	14.6	6.3
UT tract	0.3	11.9	10.3	2.9	73.5	1

The “**Phylogenetic Distribution of Genes**” is an IMG/M comparative analysis tool that provides an estimate of the phylogenetic composition of a metagenome sample based on the distribution of the best BLAST hits of the protein-coding genes in the sample. The result of “**Phylogenetic Distribution of Genes**” can be displayed using the “**Radial Phylogenetic Tree**” viewer as illustrated in Figure 2(iii), or in a tabular format consisting of a histogram with counts protein-coding genes in the sample that have best BLASTp hits to proteins of isolate genomes in each phylum or class with more than 90% identity, 60–90% identity and 30–60% identity, respectively. A specialized version of this tool, the “**Body Sites Phylogenetic Distribution**” is available on the front page of IMG/M-HMP, whereby all the genes of the metagenomic samples are grouped by their primary body site and their best blast hits against the reference genomes are organized taxonomically. The results of this comparison are displayed using the “**Radial Phylogenetic Tree**” tool with all the reference genomes organized in a color-coded hierarchical circular tree according to the taxonomic level of choice as illustrated in Figure 2(iii). Using this radial tree, the distribution of the best BLAST hits of a group of genomes or metagenomes against the reference set of genomes, can be projected. In this case, Figure 2(iii), shows the phylogenetic distribution of the genes associated with all the metagenomic samples aggregated by their primary body site, to all isolate genomes, grouped at the taxonomic level of family.

The “**Significant Family Plot**” summarizes the relationship between the samples using BLASTp-based estimation of their taxonomic composition. The tool illustrated in Figure 2(iv) is available on the front page of IMG/M-HMP. The number of genes from the sample with best BLAST hit to genes in the specific taxonomic family is used as a proxy for the abundance of microbes from this family in the sample. Only families with at least 1% contribution to the total number of genes are considered, and hierarchical clustering is performed using the gene counts described above, with the results represented as a two-dimensional dendrogram. The same results can be obtained by using the “Genome Clustering” tool and the option “**Hierarchical Clustering**” which is described below.

**Figure 3:**
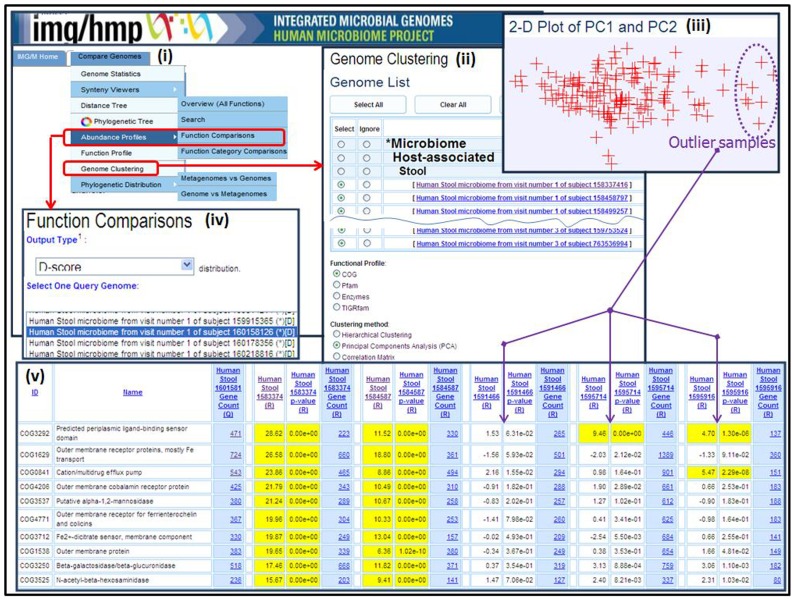
Metagenome comparison tools in IMG/M-HMP. (i) The metagenome comparison tool menu in IMG/M-HMP; (ii) Genome Clustering tool menu in IMG/M-HMP; (iii) Principal Component Analysis (PCA) plot for all stool samples using COGs showing the outlier samples; (iv) Function Comparisons tool menu in IMG/M-HMP; (v) Function Comparisons tool results using an outlier from (iii) - metagenome of stool sample of subject 160158126, visit 1 - as a query and several outlier and non-outlier samples as references.

Several other comparative analysis tools which allow examining the gene content and functional capabilities of microbial communities are available under the “**Compare Genomes**” main menu tab of IMG/M, as shown in Figure 3(i). For instance, the “**Abundance Profile Overview**” tool provides a quick estimate of the functional capabilities of metagenomes of interest in terms of the relative abundance of protein families (COGs, Pfams) and functional families (Enzymes), with the result displayed either as a heat map or in matrix format, with each column corresponding to a metagenome and each row corresponding to a family. A counterpart “**Abundance Profile Search**” tool allows finding protein families (COGs, Pfams) in metagenome datasets based on their relative abundance, with the ability of selecting abundance cut-offs and the way the results are displayed, namely using raw or normalized gene counts.

We discuss below in more detail how IMG/M HMP’s comparative analysis tools can be used in the context of a HMP specific analysis. A potential starting point for such an analysis is identifying the outliers among samples collected from the same type of human body site, such as all the human gut samples, using “**Genome Clustering**” tools, as illustrated in Figure 3(ii). In this example, Principal Component Analysis (PCA) of human gut samples based on COGs identifies several outliers, as illustrated in Figure 3(iii).

Next, the “**Function Comparison**” tool can be used to determine which protein families distinguish outlier samples from the rest of the human gut samples. The “**Function Comparison**” tool takes into account the stochastic nature of metagenome datasets and tests whether the differences in abundance of protein families can be ascribed to chance variation or not. This tool allows comparing a metagenome dataset with other metagenome datasets or reference genomes in terms of the relative abundance of protein families (COGs, Pfams, TIGRfams) and functional families (Enzymes) and assigns statistical significance to the differences in protein family abundance. In our specific example one outlier sample is selected as a query sample, and compared to reference samples that consist of both outliers and non-outliers in terms of relative abundance of COGs, as illustrated in Figure 3(iv). The result of such comparison is represented as a list of functions or protein families, whereby for each function or protein family *F*, the number of genes or an estimated gene copy number in the target (query) metagenome associated with *F* is displayed. Similar counts are displayed for each reference genome/metagenome, and the differences in protein family abundance are assessed for their statistical significance reflected in the associated **p-value** and **D-scores.** The latter represents a standard score obtained by subtracting the mean frequency of a protein family in the datasets and divided by the standard deviation of frequency of a protein family in the datasets under an assumption of normal distribution, and p-values are corrected for multiple hypothesis testing using False Discovery Rate. The cells displaying the p-value and D-score of families with statistically significant differences are highlighted in yellow. For instance, in the example shown in Figure 2(v), protein families (COGs) that are more abundant in the query sample than in non-outlier samples from Figure 3(iii) are highlighted in yellow; note that the same protein families in outlier samples from Figure 3(iii) are not highlighted in yellow, indicating that they don’t have statistically significant differences with the query sample.

The results of “**Function Comparison**” tool indicate that the outlier human gut samples shown in Figure 3(iii) may have similar functional composition. Analysis of the protein families that consistently show up as more abundant in these outlier samples, such as COG1629 (Outer membrane receptor proteins, mostly Fe transport), COG4206 (Outer membrane cobalamin receptor protein), and COG1538 (Outer membrane protein), suggests that these functional differences may be due to differences in the taxonomic composition of the samples, namely in the different abundance of Gram-positive and Gram-negative bacteria, since all of the protein families distinguishing two groups of samples are found in *Gram-negative*, but not in *Gram-positive* bacteria. Thus, it is possible that the outlier samples are dominated by *Gram-negative* bacteria, whereas non-outlier samples are dominated by *Gram-positive* bacteria. These two groups of bacteria have different surface structures, which in turn can be linked to the differences in transport mechanisms and in certain metabolic pathways.

**Figure 4:**
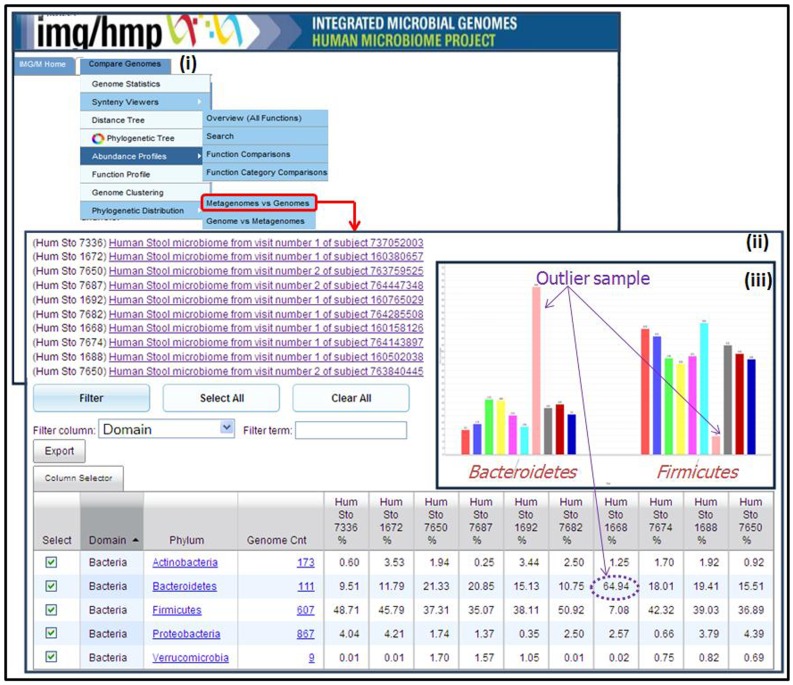
Estimation of metagenome composition using Phylogenetic Distribution tool in IMG/M-HMP. (i) Navigation to Phylogenetic Distribution tool in Compare Genomes menu in IMG/M-HMP; (ii) results of Phylogenetic Distribution comparison displayed in tabular format for one outlier sample - metagenome of stool sample of subject 160158126, visit 1– and several non-outlier samples; (iii) results of Phylogenetic Distribution comparison displayed as a bar chart with pink bars corresponding to the metagenome of stool sample of subject 160158126, visit 1.

This hypothesis can be directly tested using IMG/M-HMP tools, whereby the genes from one or more distinguishing protein families in “**Function Comparison**” results can be selected and added to “**Gene Cart”**. The scaffolds on which these genes are encoded can be added to “**Scaffold Cart**”, and analyzed in terms of BLASTp hits of all proteins encoded on them. When such analysis has been performed on the distinguishing protein families in the example above, the majority of BLASTp hits were found to be to the genomes from *Bacteroidetes* phylum, which is indeed a phylum of gram-negative bacteria. This supports the idea that functional differences between outlier and non-outlier samples are due to the differences in taxonomic composition. The “**Metagenomes Phylogenetic Distribution**” tool can be used to confirm this hypothesis, as illustrated in Figure 4(i).

The “**Metagenomes Phylogenetic Distribution**” tool is based on “**Phylogenetic Distribution of Genes**” tool described in the previous section and it provides a comparison of multiple metagenome samples based on the distribution of the best BLASTp. The results of “**Metagenomes Phylogenetic Distribution**” can be displayed in tabular format, as illustrated in Figure 4(ii) for one outlier and several non-outlier samples, which shows counts of protein-coding genes with best BLASTp hits with more than 60% identity to proteins of isolate genomes grouped by phylum. The same results can be displayed as a bar chart (Figure 4(iii)), which clearly shows that the outlier sample (pink bar) is dominated by *Bacteroidetes* (a gram-negative phylum), while non-outlier samples are dominated by *Firmicutes,* which mostly includes gram-positive bacteria.

### Availability and Future Directions

The current version of IMG/M HMP (January 2012) contains 748 metagenome datasets generated as part of the HMP initiative by sequencing samples collected from various body sites, with a total of 80 million protein coding genes. These datasets can be analyzed in the context of 6,116 bacterial, archaeal, eukaryotic, and virus reference genomes.

These samples include only assembled sequences (scaffolds/contigs) and their corresponding annotation using the HMP pipeline described at (http://hmpdacc.org/). Note that this release of IMG/M HMP contains 748 samples as opposed to the 690 samples available at the HMP-DACC website. The additional samples found in IMG/M-HMP, but not at the HMP-DACC website are those with abnormal mean contig length and CDS density. On the other hand, composite assemblies incorporating reads from different samples collected from the same body site are not included in IMG/M-HMP. In the next months IMG/M HMP will provide access to the full datasets, including unassembled sequences and body-site specific composite assemblies annotated by the standard JGI metagenome annotation pipeline.

## Methods

**IMG/M-HMP** is an IMG/M ER **data mart** focussed only on metagenome datasets produced by the US National Institute of Health (NIH) Human Microbiome Project (HMP). The entire IMG/M ER system contains about **1,741 metagenome datasets** (samples) with over **4.2 billion** protein coding **genes,** which are part of about **265 metagenome studies**, as of February 23^th^ 2012.

HMP metagenome samples are recorded in HMP’s Data Acquisition and Coordination Center (DACC) Project Catalog (http://www.hmpdacc-resources.org/hmp_catalog/), were sequenced at four genome centers (Baylor College of Medicine, Broad Institute, J. Craig Venter Institute and Washington University at St. Louis), and then processed using the SOAP denovo for assembly [20] and a MetaGenemark for predicting genes, as described in detail in [17].

Metagenome datasets are integrated with over **6,116** bacterial, archaeal, eukaryotic, and viral genomes, including **630** reference genomes sequenced as part of the HMP initiative, as well as over **110** genomes generated as part of the Genome Encyclopedia of Bacterial and Archaea Genomes (GEBA) project which aims at systematically filling the sequencing gaps along the bacterial and archaeal branches of the tree of life [21]. The reference genome baseline of IMG/M HMP also includes **1,199** plasmids and **674** genome fragments that did not come from a specific microbial genome sequencing project, and has a total of over **12.5 million** protein coding genes.
